# Blue pigmentation of neustonic copepods benefits exploitation of a prey-rich niche at the air-sea boundary

**DOI:** 10.1038/s41598-018-29869-7

**Published:** 2018-07-31

**Authors:** Janina Rahlff, Mariana Ribas-Ribas, Scott M. Brown, Nur Ili Hamizah Mustaffa, Jasmin Renz, Myron A. Peck, Kimberley Bird, Michael Cunliffe, Katharina Melkonian, Christopher J. Zappa

**Affiliations:** 1 Institute for Chemistry and Biology of the Marine Environment (ICBM), Carl von Ossietzky University Oldenburg, Schleusenstraße 1, 26382 Wilhelmshaven, Germany; 2 Lamont-Doherty Earth Observatory, Columbia University, Palisades, New York, 10964 USA; 3 German Centre for Marine Biodiversity Research, Senckenberg am Meer, Martin-Luther-King Platz 3, 20146 Hamburg, Germany; 4 Center for Earth System Research and Sustainability (CEN), University of Hamburg, Olbersweg 24, 22767 Hamburg, Germany; 5 Marine Biological Association of the United Kingdom, Plymouth, PL1 2PB UK; 6 Present Address: Group for Aquatic Microbial Ecology (GAME), University of Duisburg-Essen, Campus Essen-Biofilm Centre, Essen, Germany

## Abstract

The sea-surface microlayer (SML) at the air-sea interface is a distinct, under-studied habitat compared to the subsurface and copepods, important components of ocean food webs, have developed key adaptations to exploit this niche. By using automated SML sampling, high-throughput sequencing and unmanned aerial vehicles, we report on the distribution and abundance of pontellid copepods in relation to the unique biophysicochemical signature of the SML. We found copepods in the SML even during high exposure to sun-derived ultraviolet radiation and their abundance was significantly correlated to increased algal biomass. We additionally investigated the significance of the pontellids’ blue pigmentation and found that the reflectance peak of the blue pigment matched the water-leaving spectral radiance of the ocean surface. This feature could reduce high visibility at the air-sea boundary and potentially provide camouflage of copepods from their predators.

## Introduction

The ocean-spanning sea-surface microlayer (SML) forms the boundary between atmosphere and hydrosphere. Despite having a thickness of <1 mm, the SML has profoundly different physicochemical and biological characteristics from the underlying water (ULW)^1^. The SML provides a biogenic gelatinous framework^2^ and is typically enriched with organic matter^3^, heterotrophic microorganisms^4^ as well as higher trophic level organisms^5^.

Among zooplankton taxa living within the SML, neustonic copepods (phylum Arthropoda, class Crustacea) of the family Pontellidae have been frequently recorded in tropical regions of all oceans^6–8^. The SML is regarded as a challenging or even extreme habitat because organisms are exposed to variable temperatures and high intensities of solar and ultraviolet (UV) radiation^9^. Copepods are the most abundant metazoans on Earth^10^ and show impressive short-term adaptation to environmental stressors, e.g. downregulation of the cellular heat stress response^11^. Given their major role in marine food webs and ecosystem functioning^12^, knowledge of the tolerance limits of copepods to abiotic factors is essential if we hope to make robust projections of the effects of global change on the world’s oceans. The effects of climate-driven warming (and acidification) on the SML ecosystem and neuston-dwelling copepods, although scarcely examined to date, may be particularly dramatic.

A feature of many pontellid copepods is their blue colouring, that also occurs in other surface-dwelling mesozooplankton^13^. The colouring results from a complex of the pigment astaxanthin and a carotenoprotein^14^. Astaxanthin can be produced from dietary sources and was found to be the principal carotenoid in four different blue-pigmented copepod genera as well as in *Oikopleura dioica* of the class Appendicularia indicating convergent evolution of the feature in different neuston inhabitants^15^. Various theories have been developed to explain the significance of the blue colouring in copepods, including protection from strong solar and/or UV radiation^16,17^, camouflage against visual predators that forage in the uppermost water layers^13^ as well as recognition of conspecifics when occurring together with copepods that possess a green fluorescent protein (GFP)-based coloration^18^.

*In situ* sampling was performed on the *R/V* Falkor in the tropical Pacific Ocean, northwest of the Bismarck Sea in October and November 2016 (Supplementary Fig. S1). During daylight hours over six days, the remotely-operated Sea Surface Scanner (S^3^)^19^ was used to collect paired samples from the SML (uppermost 1 mm) and ULW (1 m reference depth). The S^3^ employs rotating glass discs^20^ to collect organisms associated with the SML. In addition, conductivity (for calculations of salinity) and temperature within the SML as well as UV radiation in air (3 m above sea level) were recorded onboard the S^3^. Spectral absorption of the water surface was measured using an unmanned aerial vehicle (UAV) and compared to the reflectance peak derived from the blue pigment of the pontellids to further investigate the camouflage hypothesis.

## Results and Discussion

**Figure 1:**
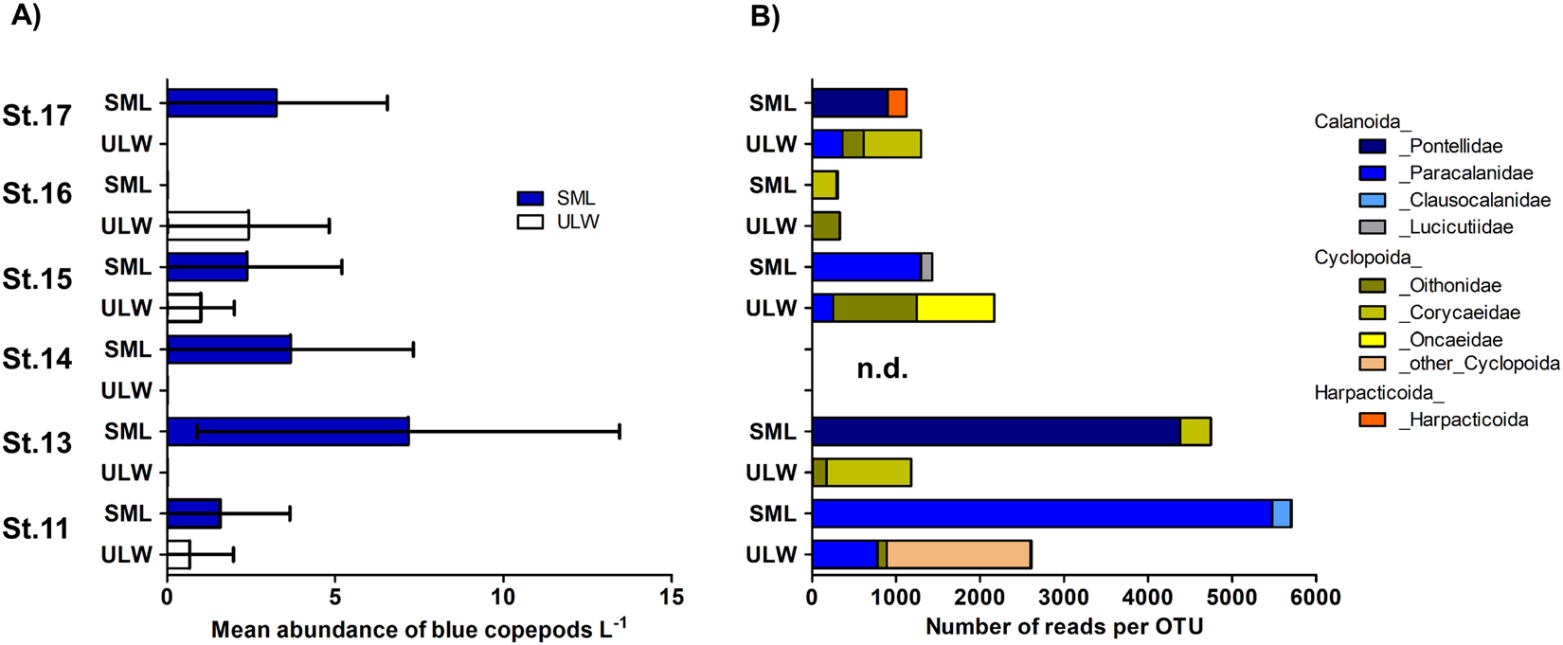
Copepod distribution at the air-sea boundary. (**A**) Mean (±standard deviation) of whole pontellid copepods L^−1^ in SML and ULW. (**B**) Number of reads per OTU assigned for different copepod orders and families from SML and ULW (1 m depth). Stations as in (**A**). N.d. = not determined, SML = sea-surface microlayer, ULW = underlying water, OTU = operational taxonomic unit, St = Station.

Blue copepods (prosoma length of ~2 mm) collected with the S^3^ were counted and identified to the calanoid copepod family Pontellidae in subsamples from stations 11, 13, 14 and 17 (Supplementary Fig. S1). More precisely, *Ivellopsis denticauda* was found at station 11, whereas *Pontella fera* occurred at stations 13, 14, 17 (Supplementary Fig. S1). Besides the blue pontellids no other copepods were observed in both SML and ULW samples. Neustonic copepods were previously found to be enriched (10–100x) in the uppermost 0–5 cm layer of the ocean, compared to the ULW^21^. Using the S^3^ we found higher abundance of pontellids in the SML (the uppermost 1 mm) compared to the ULW at one meter (Fig. 1A). High-throughput sequencing of the total eukaryote 18S ribosomal RNA (rRNA) encoding genes on additional manually-collected SML and ULW samples confirmed predominant SML enrichment for the order Calanoida including the families Pontellidae and Paracalanidae, whereas different copepods of the order Cyclopoida were most prevalent in the ULW (Fig. 1B). Using BLAST analysis, we found that operational taxonomic units (OTUs) from SML samples shared high identity with the blue pontellids *Pontella fera* (100%) and *Anomalocera patersoni* (97%) at two stations (Supplementary Table S1). Due to the lack of reference sequences for copepod species in the Genbank and SILVA database, the true copepod community composition could not be reliably determined. While *Anomalocera patersoni* (97%) was the closest hit in the database, morphological analysis indicating presence of *Ivellopsis denticauda* might provide the better estimate for the true identity of the species. Although pontellids were previously noted in the SML via sampling with a Nytex screen^3^ or by sampling of the upper 10 cm using a neuston catamaran^8^, this is the first study to link their enrichment at the immediate air-sea boundary with physical characteristics, i.e., salinity and temperature of the SML measured during sampling and with the availability of food, i.e. chlorophyll *a* (chl *a*) as an indicator for autotrophic biomass.

When data were pooled across all five days and stations containing pontellids in the SML (Fig. 1A), the peak abundance occurred at 30.7 °C and a salinity of 33.8 (Supplementary Fig. S2). SML and ULW temperature and salinity were significantly different from each other at most stations (Supplementary Fig. S3a,b, Supplementary Table S2). However, neither SML temperature nor salinity being recorded simultaneously to copepod sampling were significantly correlated to copepod abundance in SML samples (Supplementary Fig. S4a,b, Supplementary Table S3). Previous studies on *Pontella fera* in the South Pacific reported increased abundance in waters >28.5 °C and at salinity <34.5^22^, and that this species can be abundant in surface waters during daytime^7^. Our data on copepod presence in the SML match the known abiotic preferences (Supplementary Fig. S2). Compared to the ULW, our data indicate that the SML was often more saline (mean ± standard deviation (STD) difference = 0.3 ± 0.5, n = 95) likely due to evaporation and also colder (mean ± STD difference = −0.3 ± 0.2 °C, n = 108), likely due to its heat flux^23^ producing a “cool skin effect”. Due to the given profiles of temperature and salinity, the SML is denser compared to the ULW, but its stability and buoyancy is retained by the forces of interfacial tension between SML and ULW and by the surface tension of the SML. These physical characteristics of the SML support the establishment of a unique set of organisms^24^ and may be especially advantageous to survival in the tropics. For instance, only at station 16 SML temperature was higher than its ULW counterpart (Supplementary Fig. S3a) and only at this station blue copepods were absent from the SML (Fig. 1A,B).

**Figure 2:**
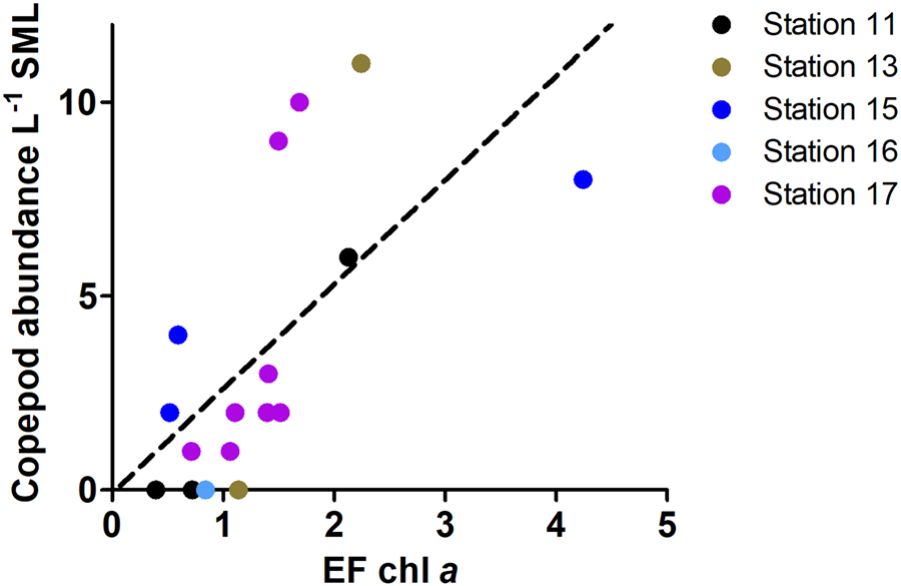
Pontellid abundance in relation to autotrophic biomass. Number of pontellid copepods in SML versus enrichment factor (EF) for chlorophyll *a* (chl *a*), i.e. the ratio of chl *a* concentration in SML over ULW for individual sampling bottles pooled across all stations except for station 14 (no chl *a* readings).

Additional advantages of living at the immediate air-sea interface include the ability to escape predation by leaping out of the water^25,26^ and the ability to reduce energy costs for routine locomotion in this physically stable niche. Surface tension and light availability also favor enrichment of autotrophic biomass (chl *a*) in the SML over ULW. Chl *a* concentration was not significantly different between SML and ULW over all stations (Supplementary Table S4, Supplementary Fig. S5, Mann-Whitney-U-test, U value = 103, p = 0.16, *n* = 17), however its enrichment in the SML over ULW was positively and significantly correlated with blue copepod abundance in the SML (Fig. 2, Spearman rank correlation coefficient = 0.70, p = 0.0017, *n* = 17). High enrichment of chl *a* -bearing microalgae in the SML over ULW supports suspension feeding by calanoids and, thus, might explain the preference of pontellid copepods to inhabit the SML. It should be noted that enrichment factors (EF) in this dataset (Fig. 2) are even underestimated, because samples for chl *a* originated from the same bottles copepods were counted from, meaning that algal biomass in these samples was already depleted by copepod grazing.

**Figure 3:**
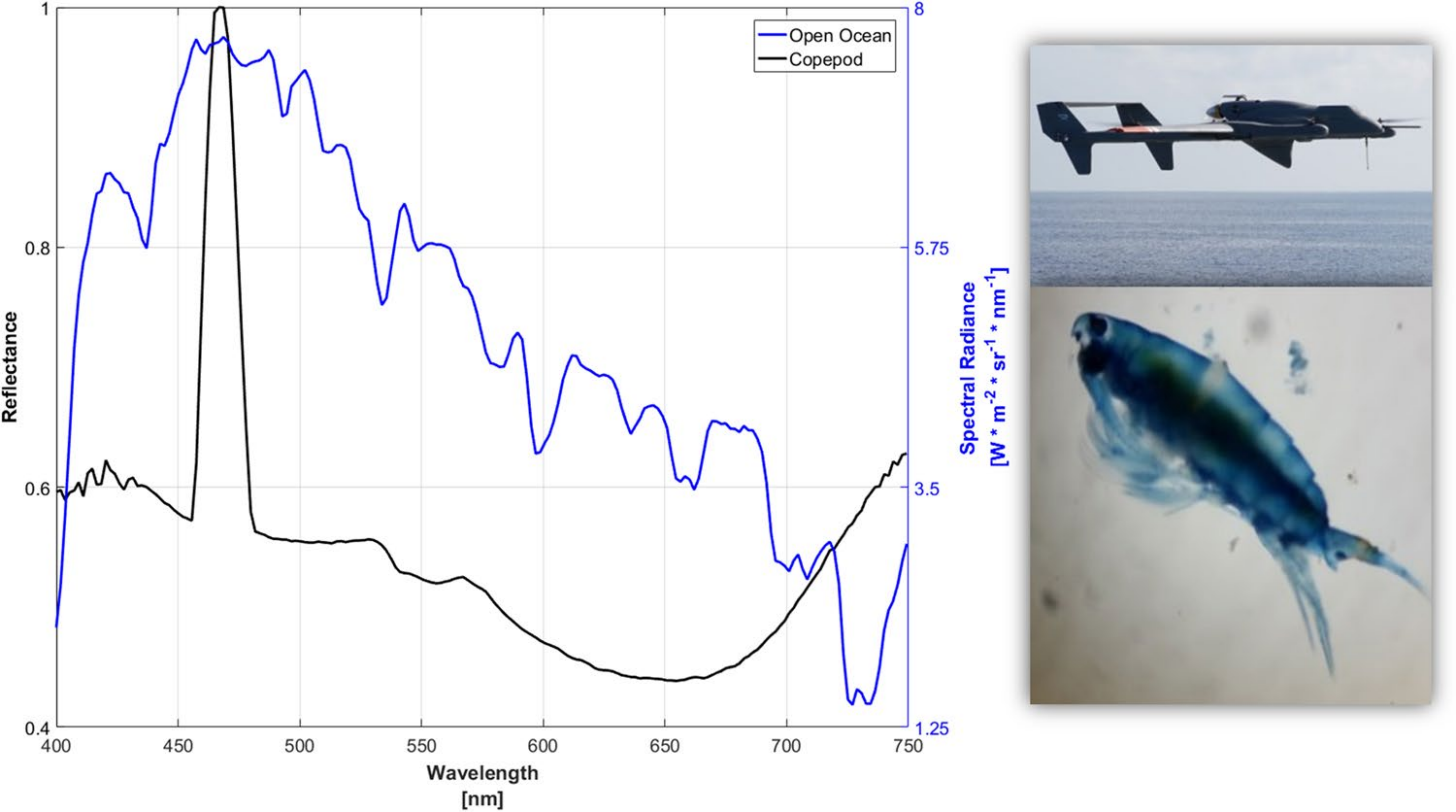
Reflectance spectrum of ocean surface and pontellid copepods. The water-leaving spectral radiance of the ocean surface measured by a hyperspectral visible imaging spectrometer aboard the ship-deployed unmanned aerial vehicle (UAV, upper picture) flying over the S^3^ during the sampling of the pontellid copepods (lower picture). The same hyperspectral visible imaging spectrometer was used in the *R/V* Falkor’s laboratory to measure the reflectance spectrum of copepod pigments.

Hyperspectral imaging of the reflectance spectra of the copepod pigment and of the ocean surface water-leaving radiance measured from the UAV are shown in Fig. 3. The spectral peak of the copepod pigment, measured directly after SML sampling, was 466.8 to 468.7 nm with a bandwidth of roughly 455.6 to 479.8 nm. The sharp, narrow spectral peak of the copepod pigment is in striking contrast to the broad absorption spectrum of water-extracted solutions of this blue pigment reported by Herring^16^. The reflectance peak of the copepod pigment measured onboard the *R/V* Falkor lies within the maximum water-leaving radiance spectrum from the ocean surface. The astaxanthin pigment in these copepods, thus, might provide effective camouflage, potentially reducing risks of visual detection by predators in the SML and/or the ULW^13^. While pigmented copepods generally experience a higher predation risk compared to transparent individuals^27^, some blue copepods such as *Pontella mimocerami* can additionally exhibit green fluorescence serving a potential role in counter-shading, a mechanism being analogous to bioluminescence^28^. This might in turn aid the crustaceans to escape predation during the night, e.g. during diel vertical migration. Whether the pontellids we observed at the air-sea interface in the open ocean also expressed GFP-like proteins remains however to be determined.

Moreover, the carotenoid pigment appears to provide protection against UV radiation^29^ by holding antioxidant properties and scavenging reactive oxygen species^17^. The abundance of copepods collected with the S^3^ seemed unrelated to an index of UV irradiance, i.e. pontellids remained present in the SML at very high UV index values (i.e. ≥10, Supplementary Fig. S6) and no enhanced mortality, i.e. increased numbers of carcasses could be observed in our samples being in contrast to other recent findings^30^. Apart from surface avoidance behavior, a typical response of copepods to UV stress^31^, and possessing sun screening carotenoids such as astaxanthin, emerging work has shown that UV-protective mycosporine-like amino acids (MAA) have an important role in UV protection of near-surface (top 50 cm) zooplankton communities^32^. Whether pontellid copepods inhabiting the SML rely on MAA in addition to their carotenoid-protein-complex still remains to be determined. At least, the SML and therein accumulating algal biomass might provide a good basis for maintaining both strategies of photo-protection because the synthesis of carotenoids, e.g. astaxanthin from β-carotene, and MAAs is dependent on the availability of algal precursor molecules^15,33^. While recent work has shown that not only UV radiation and fish predation can affect copepod pigmentation, stress responses expressed by colouring can also vary between sex and life-stage of the copepod^34^ which we could however not consider in the given study.

Pontellid copepods have a unique life history strategy in the tropical ocean shaped by their ability to tolerate and exploit a prey-rich and slightly cooler SML environment of high physical stability. While being inhospitable to most other meso-zooplankton, pontellids most likely benefit from their pigmentation and adaptive behavior^25,35^ to cope with the disadvantages of high visibility and increased solar and UV radiation at the air-sea interface.

## Methods

### Copepod sampling via the S^3^

Copepods were collected by the S^319^ over a distance of 5–7 km, with an average sampling speed of 2 km h^−1^ at a minimum distance of 100 m from the *R/V* Falkor. Special neuston nets can collect organisms in the 0–5 cm layer^36^ but, as only the hydrophobic SML adheres to the glass discs, the S^3^ can collect copepods exclusively from the uppermost 1 mm. Sampled volumes are shown in Supplementary Table S5. Copepod subsamples from most stations were later identified by light microscopy (Supplementary Table S6). Water from 1 m was pumped up by the S^3^ for reference purposes. Twelve pairs of 1-L bottles were filled with SML and ULW samples during each sampling day, and each bottle filling took approximately 2–3 minutes. Operation of the S^3^ was between 11 pm and 7 am UTC time, which corresponds to local daytime. Temperature, conductivity and UV index were measured onboard the S^3^ as previously described^19^. Salinity was computed from the conductivity and temperature data using algorithms of the fundamental properties of seawater^37^. Measurements of temperature and salinity were taken from the sensor measurements onboard the S^3^ conducted at the same time copepods were sampled. At station 16 salinity from the ULW was not properly recorded due to sensor issues and thus omitted from the data set. The UV index was measured on the mast of the S^3^, approximately 3 m above the sea surface, and ranged from 0 to 11 (an index of 10 is equal to an Erythemal Action Spectrum (EAS) weighted irradiance of 0.25 W m^−2^).

### Chlorophyll *a* analysis

Water from random SML and ULW pairs of 1-L bottles was taken for discrete chlorophyll *a* analysis. The fluorometer (JENWAY 6285, Bibby Scientific Ltd., UK) was calibrated before measurements. Readings on standards were taken by using pure chl *a* extracted from spinach (Sigma Aldrich, Germany). Water samples (600–800 mL) were filtered immediately onto glass microfiber filters (GF/F, diameter: 25 mm, Whatman, UK) and were stored at −20 °C for further analysis for up to 4 weeks. The filtered samples were then extracted in 3 mL of 90% ethanol solution for 24 hours and in dark condition, before being measured fluorometrically according to the EPA Method 445.0^38^. The enrichment factor (EF) gives the ratio of the chl *a* concentration in the SML to its ULW counterpart from paired sampling bottles (SML and ULW) derived from stations 11 (*n* = 3), 13 (*n* = 2), 15 (*n* = 3), 16 (*n* = 1), and 17 (*n* = 8). An EF >1 indicates an enrichment of chl *a* within the SML, whereas EF < 1 means a depletion.

### High-throughput sequencing

In addition, manual glass plate sampling^39^ from a small boat and Illumina MiSeq sequencing on the 18S rRNA gene was used to determine the copepod community composition. Sampling was performed shortly before and after S^3^ operation and the normalised abundance per operational taxonomic unit (OTU) pooled for each station 11, 13, 15, 16, 17. Seawater samples (500 mL) from the SML and ULW were filtered onto 0.2 µm cellulose nitrate membranes (Whatman, UK), and DNA was extracted using a commercially available DNeasy kit (Qiagen, UK). Primers used for targeting the V4 region of the 18S rRNA gene were 572 F and 1009R^40^. 18S rRNA encoding gene library preparation and sequencing were performed at the Integrated Microbiome Resource (IMR) at the Centre for Comparative Genomics and Evolutionary Bioinformatics (CGEB), Dalhousie University as previously conducted^41^. Sequences were processed in QIIME v1.9.1^42^ and USEARCH v9^43^ as described elsewhere^44^. OTUs assigned as Copepoda (SILVA 128 database) were filtered from the 18S rRNA data set. Phylogenetic affiliations were determined using the Basic Local Alignment Search Tool (https://blast.ncbi.nlm.nih.gov/Blast.cgi).

### UAV operation and spectral analyses

During the cruise, unmanned aerial vehicles (UAVs, Latitude Engineering model HQ-60) were flown. They carried a down-looking Headwall Photonics model Micro-Hyperspec A-Series VNIR hyperspectral visible (400–1000 nm) imaging spectrometer with better than 3 nm spectral resolution for water-leaving spectral radiance measurements to determine ocean color and biogeochemical mapping. The spectrometer measurements from the UAV were performed directly over the ocean surface sampled by the S^3^, and the open ocean spectrum in Fig. 3 is an average over 1 km or 1800 spectra. The same hyperspectral imaging spectrometer was used in the *R/V’s* laboratory to measure copepod pigments on homogenate of six crushed individuals. The copepod mixture was prepared on a glass slide and illuminated by a diffuse white light source. For the copepod reflectance spectrum, we divided the average of 725 spectra of the copepod mixture by the source spectrum.

### Statistical analysis

Non-parametric Spearman rank correlation analysis (two-tailed, 95% confidence level) for EF of chl *a*, temperature and salinity versus copepod abundance in SML was performed. Chl *a*, temperature and salinity differences between SML and ULW were analyzed by means of a two-tailed Mann-Whitney U test. A non-parametric approach was chosen due to the limited number of observations that made it difficult to assess normality and homoscedasticity of the data. All analyses were carried out using GraphPad Prism (v5.00 GraphPad Software, San Diego CA, USA).

### Data availability

The datasets generated during and/or analysed during the current study are available from the corresponding author on reasonable request.

## Electronic supplementary material

Supplementary material

## References

[1] Engel, A. *et al*. The ocean’s vital skin: toward an integrated understanding of the sea surface microlayer. *Front. Mar. Sci.* **4**, 10.3389/fmars.2017.00165 (2017).

[2] Wurl O, Holmes M (2008). The gelatinous nature of the sea-surface microlayer. Mar. Chem..

[3] Sieburth JM (1976). Dissolved organic matter and heterotrophic microneuston in the surface microlayers of the North Atlantic. Science.

[4] Franklin MP (2005). Bacterial diversity in the bacterioneuston (sea surface microlayer): the bacterioneuston through the looking glass. Environ. Microbiol..

[5] Brodeur RD (1989). Neustonic feeding by juvenile salmonids in coastal waters of the Northeast Pacific. Can. J. Zool..

[6] Heinrich AK (1971). On the near-surface plankton of the eastern South Pacific Ocean. Mar. Biol..

[7] Heinrich AK (2010). Influence of the monsoon climate on the distribution of neuston copepods in the Northeastern Indian Ocean. Oceanology.

[8] Turner JT, Collard SB, Wright JC, Mitchell DV, Steele P (1979). Summer distribution of pontellid copepods in the neuston of the Eastern Gulf of Mexico continental-shelf. B. Mar. Sci..

[9] Maki JS (2003). Neuston microbiology: life at the air-water interface. Encyclopedia of Environmental Microbiology.

[10] Humes, A. G. How many copepods? In *Ecology and Morphology of Copepods*. *Developments in Hydrobiology* (eds Ferrari F.D. and Bradley B.P.) Vol. 102 Springer, Dordrecht, 1–7 (1994).

[11] Rahlff J (2017). Short-term molecular and physiological responses to heat stress in neritic copepods *Acartia tonsa* and *Eurytemora affinis*. Comp. Biochem. Physiol. A, Mol. Integr. Physiol..

[12] Mauchline, J. The biology of calanoid copepods. In *Advances in Marine Biology* (eds Blaxter, J.H.S, Southward, A.J. and Tyler, P.A.) Vol. 33 Academic Press, San Diego, 1–702 (1998).

[13] Herring PJ (1967). The pigments of plankton at the sea surface. Symp. Zool. Soc. Lond.

[14] Zagalsky P, Herring PJ (1972). Studies on a carotenoprotein isolated from the copepod, *Labidocera acutifrons* and its relationship to the decapod carotenoproteins and other polyene-binding proteins. Comp. Biochem. Physiol. B, Biochem. Mol. Biol..

[15] Mojib N (2014). Carotenoid metabolic profiling and transcriptome‐genome mining reveal functional equivalence among blue‐pigmented copepods and appendicularia. Mol. Ecol..

[16] Herring PJ (1965). Blue pigment of a surface-living oceanic copepod. Nature.

[17] Caramujo M-J, de Carvalho CC, Silva SJ, Carman KR (2012). Dietary carotenoids regulate astaxanthin content of copepods and modulate their susceptibility to UV light and copper toxicity. Mar. Drugs.

[18] Shagin DA (2004). GFP-like proteins as ubiquitous metazoan superfamily: evolution of functional features and structural complexity. Mol. Biol. Evol..

[19] Ribas-Ribas M, Mustaffa NIH, Rahlff J, Stolle C, Wurl O (2017). Sea Surface Scanner (S): a catamaran for high-resolution measurements of biogeochemical properties of the sea surface microlayer. J. Atmospheric Ocean. Technol..

[20] Shinki M, Wendeberg M, Vagle S, Cullen JT, Hore DK (2012). Characterization of adsorbed microlayer thickness on an oceanic glass plate sampler. Limnol. Oceanogr. Meth..

[21] Zaitsev, Y. In *The Sea Surface and Global Change* (eds Liss, P. S. & Duce, R. A.) 371–382 (Cambridge University Press, 2005).

[22] Sherman K (1964). Pontellid copepod occurrence in the central South Pacific. Limnol. Oceanogr..

[23] Saunders PM (1967). The temperature at the ocean-air interface. J. Atmospheric Sci..

[24] Hardy JT (1982). The sea surface microlayer: biology, chemistry and anthropogenic enrichment. Progr. Oceanogr..

[25] Gemmell BJ, Jiang H, Strickler JR, Buskey EJ (2012). Plankton reach new heights in effort to avoid predators. Proc. Biol. Sci..

[26] Svetlichny L, Larsen PS, Kiørboe T (2018). Swim and fly: escape strategy in neustonic and planktonic copepods. J. Exp. Biol..

[27] Gorokhova E, Lehtiniemi M, Motwani NH (2013). Trade-offs between predation risk and growth benefits in the copepod *Eurytemora affinis* with contrasting pigmentation. Plos One.

[28] Hunt ME, Scherrer MP, Ferrari FD, Matz MV (2010). Very bright green fluorescent proteins from the pontellid copepod *Pontella mimocerami*. Plos One.

[29] Byron ER (1982). The adaptive significance of calanoid copepod pigmentation - a comparative and experimental-analysis. Ecol..

[30] Mojib N, Thimma M, Kumaran M, Sougrat R, Irigoien X (2017). Comparative metatranscriptomics reveals decline of a neustonic planktonic population. Limnol. Oceanogr..

[31] Alonso C, Rocco V, Barriga JP, Battini MA, Zagarese H (2004). Surface avoidance by freshwater zooplankton: field evidence on the role of ultraviolet radiation. Limnol. Oceanogr..

[32] Fileman ES (2017). Stress of life at the ocean’s surface: latitudinal patterns of UV sunscreens in plankton across the Atlantic. Prog. Oceanogr..

[33] Hylander S, Jephson T (2010). UV protective compounds transferred from a marine dinoflagellate to its copepod predator. J. Exp. Mar. Biol. Ecol..

[34] Brüsin M, Svensson PA, Hylander S (2016). Individual changes in zooplankton pigmentation in relation to ultraviolet radiation and predator cues. Limnol. Oceanogr..

[35] Manor S, Polak O, Saidel WM, Goulet TL, Shashar N (2009). Light intensity mediated polarotaxis in *Pontella karachiensis* (Pontellidae, Copepoda). Vision Research.

[36] Zaitsev, Y. P. *Marine Neustonology* (translated from Russian). National Marine Fisheries Service, NOAA and National Science Foundation, National Technical Information Service, Springfield, Virginia (1971).

[37] Fofonoff, N. & Millard, R. Jr. Algorithms for computation of fundamental properties of seawater. Endorsed by Unesco/SCOR/ICES/IAPSO Joint Panel on Oceanographic Tables and Standards and SCOR Working Group 51. Unesco Technical Papers in Marine Science, No. 44. (1983).

[38] Arar, E. J. & Collins, G. B. Method 445.0: *In vitro* determination of chlorophyll a and pheophytin a in marine and freshwater algae by fluorescence. (United States Environmental Protection Agency, Office of Research and Development, National Exposure Research Laboratory Cincinnati, 1997).

[39] Harvey GW, Burzell LA (1972). A simple microlayer method for small samples. Limnol. Oceanogr..

[40] Comeau AM, Li WK, Tremblay JE, Carmack EC, Lovejoy C (2011). Arctic Ocean microbial community structure before and after the 2007 record sea ice minimum. Plos One.

[41] Comeau AM, Douglas GM, Langille MG (2017). Microbiome helper: a custom and streamlined workflow for microbiome research. mSystems.

[42] Caporaso JG (2010). QIIME allows analysis of high-throughput community sequencing data. Nat. Methods.

[43] Edgar RC (2010). Search and clustering orders of magnitude faster than BLAST. Bioinformatics.

[44] Taylor JD, Cunliffe M (2014). High-throughput sequencing reveals neustonic and planktonic microbial eukaryote diversity in coastal waters. J. Phycol..

