# Supplementary material for: Blue pigmentation of neustonic copepods benefits exploitation of a prey-rich niche at the air-sea boundary
Source: Sci Rep. 2018 Jul 31;8:11510. doi: 10.1038/s41598-018-29869-7 (PMC6068160; doi:10.1038/s41598-018-29869-7)
Supplement: Supplementary file 1 — Supplementary material [file 41598_2018_29869_MOESM1_ESM.pdf]

Supplementary Material

Blue pigmentation of neustonic copepods benefits exploitation of a prey-rich niche at the air-sea boundary

Janina Rahlff[1*], Mariana Ribas-Ribas[1*], Scott M. Brown[2], Nur Ili Hamizah Mustaffa[1], Jasmin Renz[3], Myron A. Peck[4], Kimberley Bird[5], Michael Cunliffe[5], Katharina Melkonian[1], Christopher J. Zappa[2]

[1]Institute for Chemistry and Biology of the Marine Environment (ICBM), Carl von Ossietzky University Oldenburg, Schleusenstraße 1, 26382, Wilhelmshaven, Germany

[2]Lamont-Doherty Earth Observatory, Columbia University, Palisades, New York, 10964, USA

[3]Forschungsinstitut Senckenberg, German Centre for Marine Biodiversity Research (DZMB), Martin-Luther-King Platz 3, 20146 Hamburg, Germany

[4]Center for Earth System Research and Sustainability (CEN), University of Hamburg, Olbersweg 24, 22767 Hamburg, Germany

[5]Marine Biological Association of the United Kingdom, Plymouth PL1 2PB, UK

*Corresponding authors:

<Janina.rahlff@uni-due.de>, Present address: Group for Aquatic Microbial Ecology (GAME), University of Duisburg-Essen, Campus Essen-Biofilm Centre, Essen, Germany

<Mariana.ribas.ribas@uni-oldenburg.de>

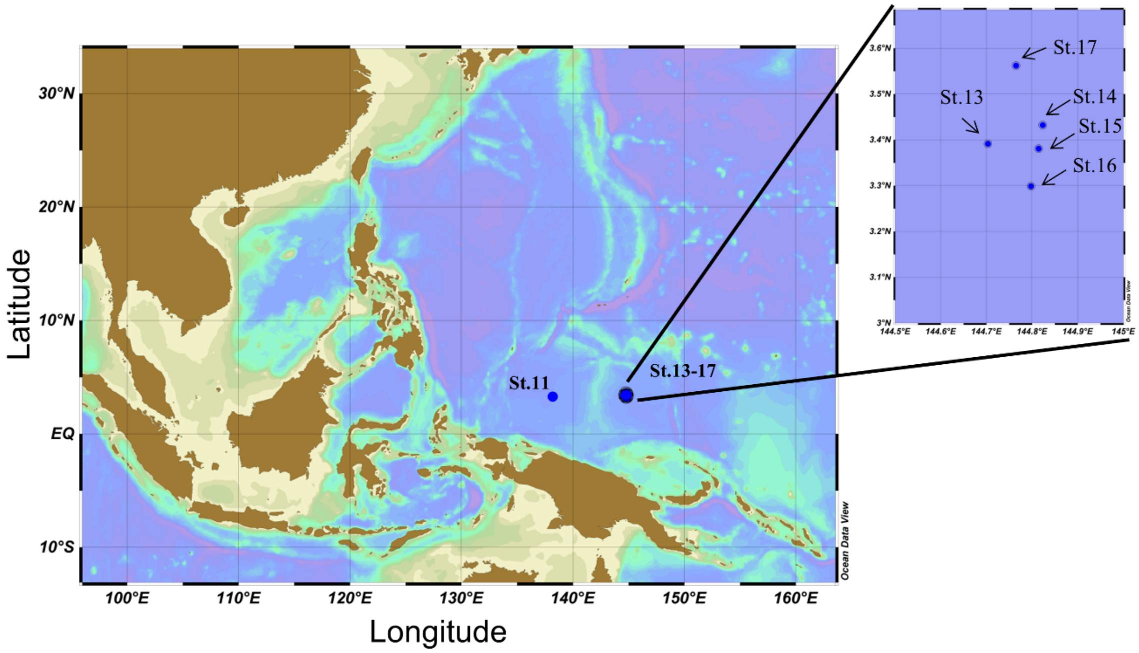

Supplementary Figure S1: Station map for S^3 copepod sampling [1].

Supplementary Table S1: Copepod OTUs found in sea-surface microlayer (SML; min. 10 sequence reads per OTU) of the Pacific Ocean. Samples are derived from manual glass plate sampling. No manual sampling took place at Station 14. BLAST= Basic Local Alignment Search Tool

Order	Station	No. of reads in SML	Phylogenetic affiliation (BLAST)	% identity	% query cover	e value	Accession number	Family	OTU#	Genbank Accession number (this study)
Calanoida	11	5476	*Acrocalanus gracilis*	100%	100%	0	JQ911938.1	Paracalanidae	SML_OTU#5	MG811792
		221	*Clausocalanus arcuicornis*	100%	100%	0	HM997079.1	Clausocalanidae	SML_OTU#96	MG811798
	13	3638	*Anomalocera patersoni*	97%	100%	0	JX995305.1	Pontellidae	SML_OTU#22	MG811795
		742	*Pontella fera*	100%	100%	0	GU969189.1	Pontellidae	SML_OTU#33	MG811796
	15	1290	*Delibus* sp.	99%	100%	0	JQ911952.1	Paracalanidae	SML_OTU#14	MG811793
		131	*Lucicutia flavicornis*	100%	100%	0	HM997055.1	Lucicutiidae	SML_OTU#228	MG811801
	17	896	*Pontella fera*	100%	100%	0	GU969189.1	Pontellidae	SML_OTU#33	MG811796
Cyclopoida	11	11	*Cyclopina* sp.	95%	100%	2E-172	MF077741.1	Cyclopinidae	SML_OTU#18	MG811794
	13	361	*Corycaeus speciosus*	100%	100%	0	GU969165.1	Corycaeidae	SML_OTU#64	MG811797
	16	289	*Corycaeus speciosus*	100%	100%	0	GU969165.1	Corycaeidae	SML_OTU#64	MG811797
		16	*Anthessius* sp.	95%	100%	3E-169	AY627002.1	Anthessiidae	SML_OTU#937	MG811802
Harpacticoida	17	175	*Miracia efferata*	99%	100%	0	EU380294.1	Miraciidae	SML_OTU#103	MG811799
		53	*Superornatiremidae* sp.	85%	100%	1E-117	MF077702.1	Superornatiremidae	SML_OTU#215	MG811800

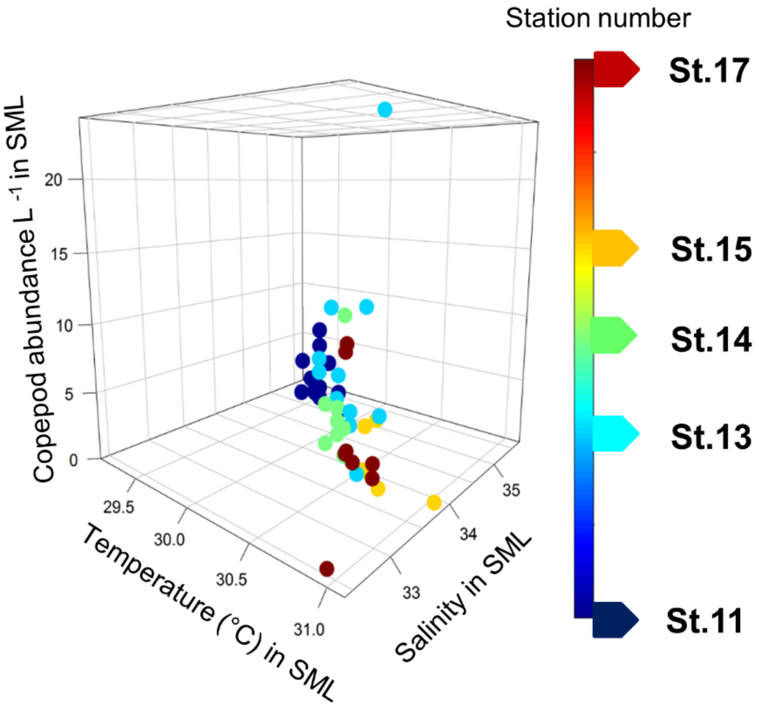

Supplementary Figure S2: Abundance of pontellid copepods in the sea-surface microlayer (SML) versus salinity and temperature of the SML.

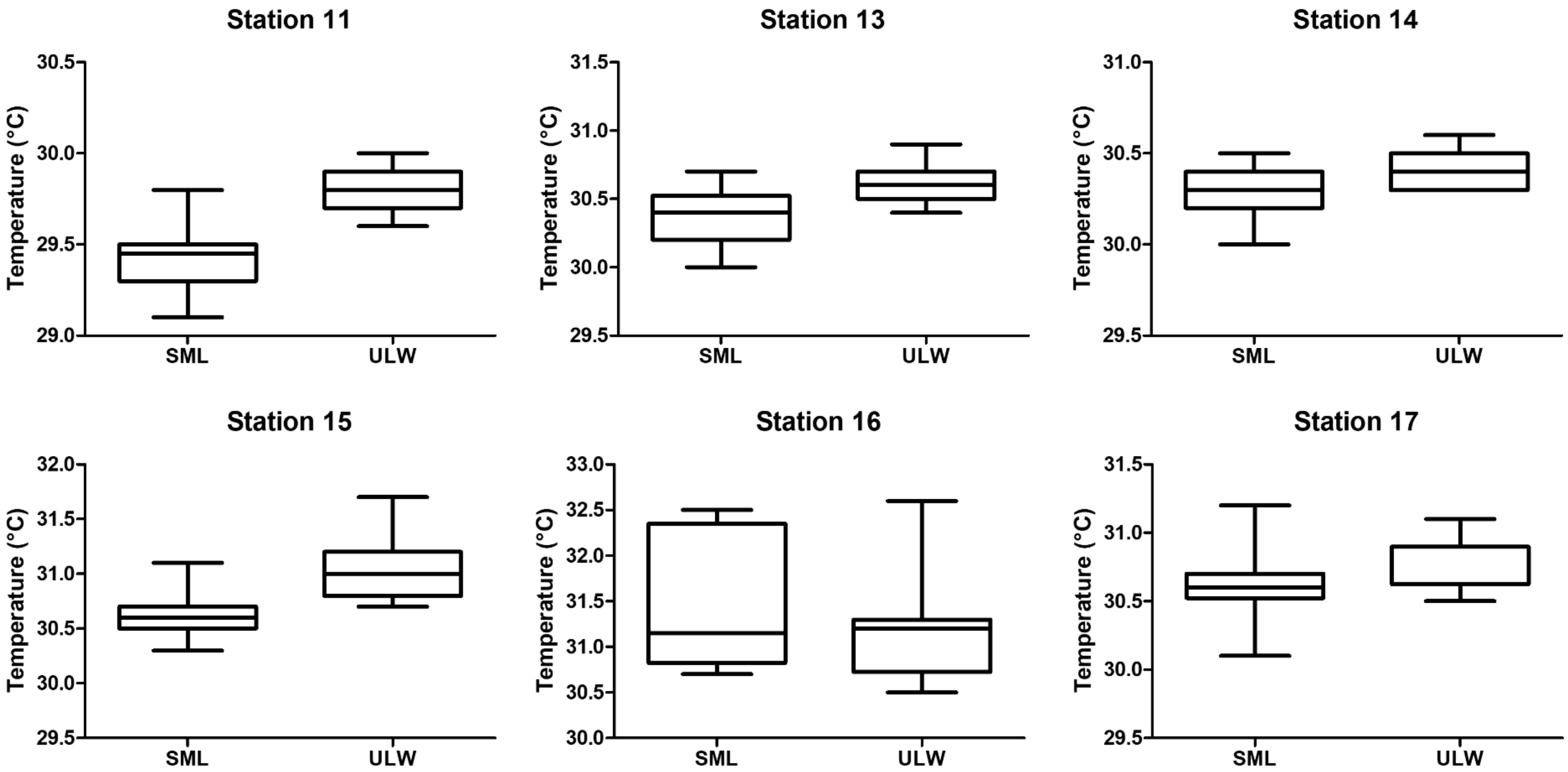

Supplementary Figure S3a: Comparison of SML and ULW temperature for stations 11 and 13-17. Whiskers indicate min. to max. values

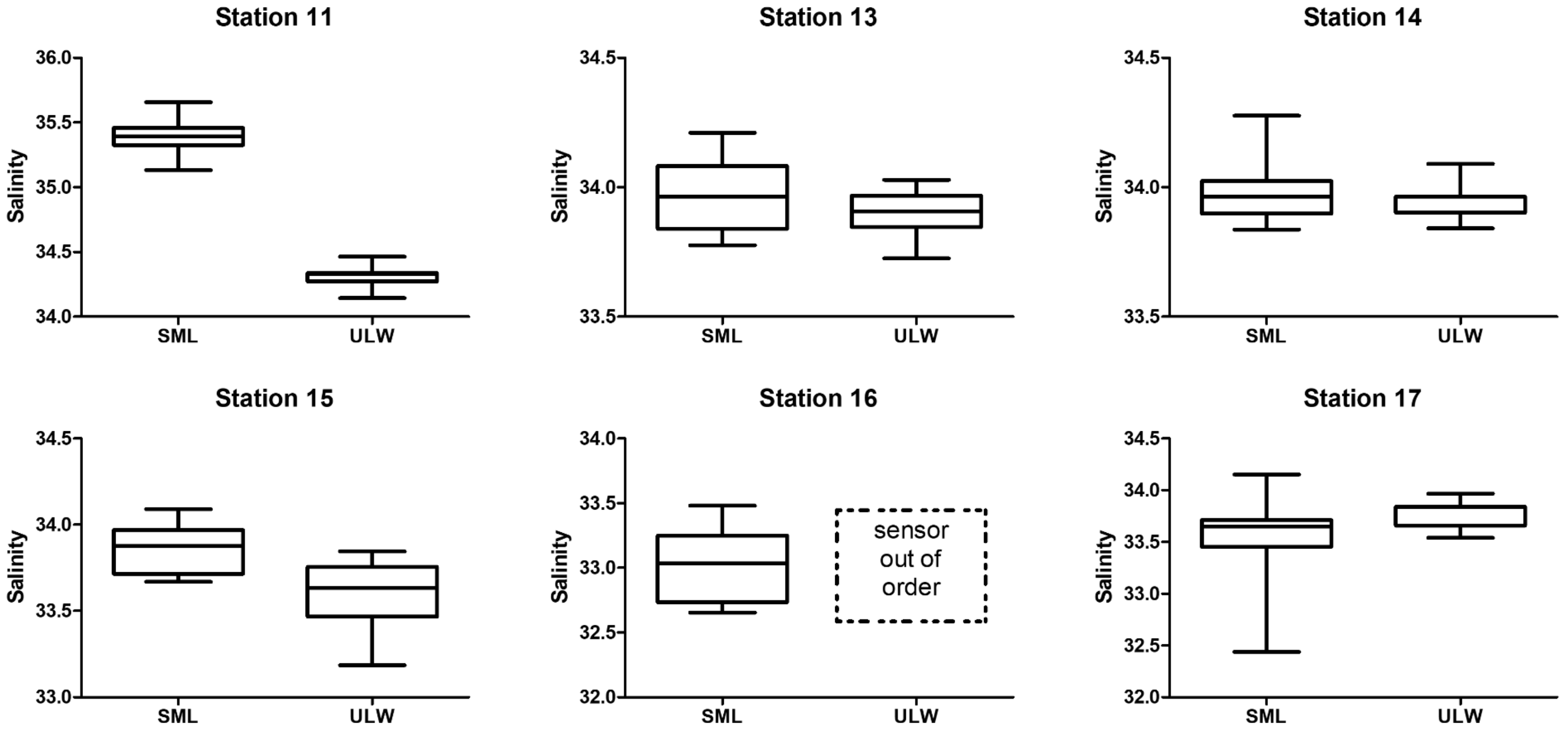

Supplementary Figure S3b: SML and ULW salinity for stations 11 and 13-17. ULW salinity for station 16 is missing due to sensor issues. Whiskers indicate min. to max. values.

Supplementary Table S2: Results from non-parametric Mann-Whitney U tests for comparisons of temperature and salinity for SML and ULW. n.d.=not determined, n.s.=not significant

	Station number	11	13	14	15	16	17	All stations
**Temperature (SML vs ULW)**	Number of observations	24	22	22	15	24	24	132
	Mann-Whitney U value	19.5	80.5	119	19	222	132	6755
	p-value	<0.0001	0.0001	0.0028	0.0001	0.1748	0.0011	0.016
	Significance	***	***	**	***	n.s.	**	**
**Salinity (SML vs ULW)**	Number of observations	24	22	17	14	n.d.	17	94
	Mann-Whitney U value	0	182	144.5	27.5	n.d.	89	3709
	p-value	<0.0001	0.1623	0.9861	0.0013	n.d.	0.0581	0.0573
	Significance	***	n.s.	n.s.	**	n.d.	n.s.	n.s.
**Chlorophyll a (SML vs ULW)**	Number of observations	n.d.	n.d.	n.d.	n.d.	n.d.	n.d.	17
	Mann-Whitney U value	n.d.	n.d.	n.d.	n.d.	n.d.	n.d.	103
	p-value	n.d.	n.d.	n.d.	n.d.	n.d.	n.d.	0.1579
	Significance	n.d.	n.d.	n.d.	n.d.	n.d.	n.d.	n.s.

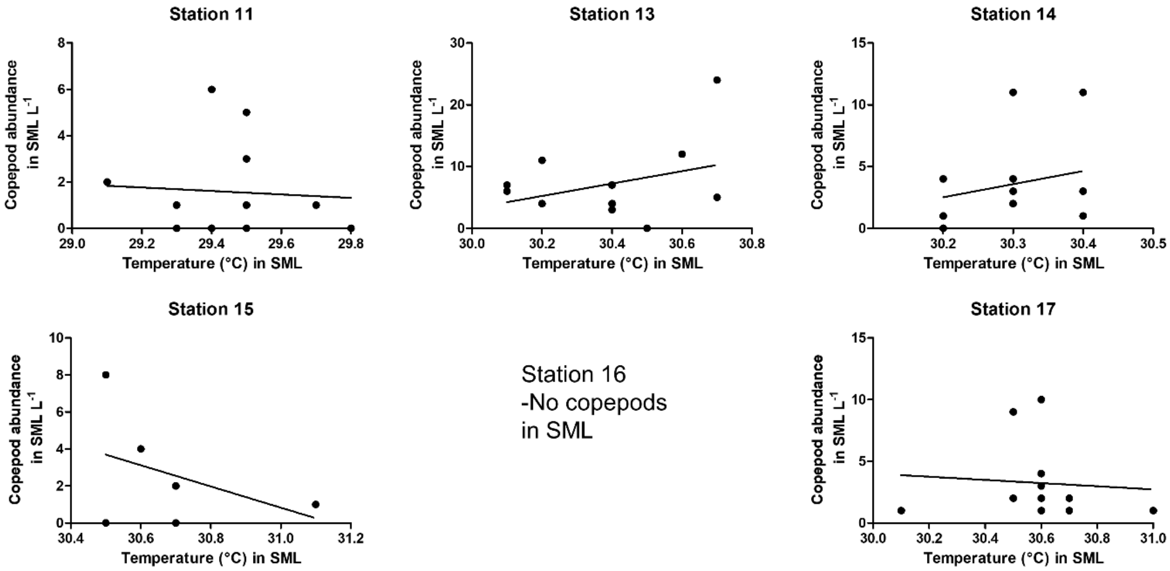

Supplementary Figure S4a: Relation of neustonic copepod abundance and temperature in SML.

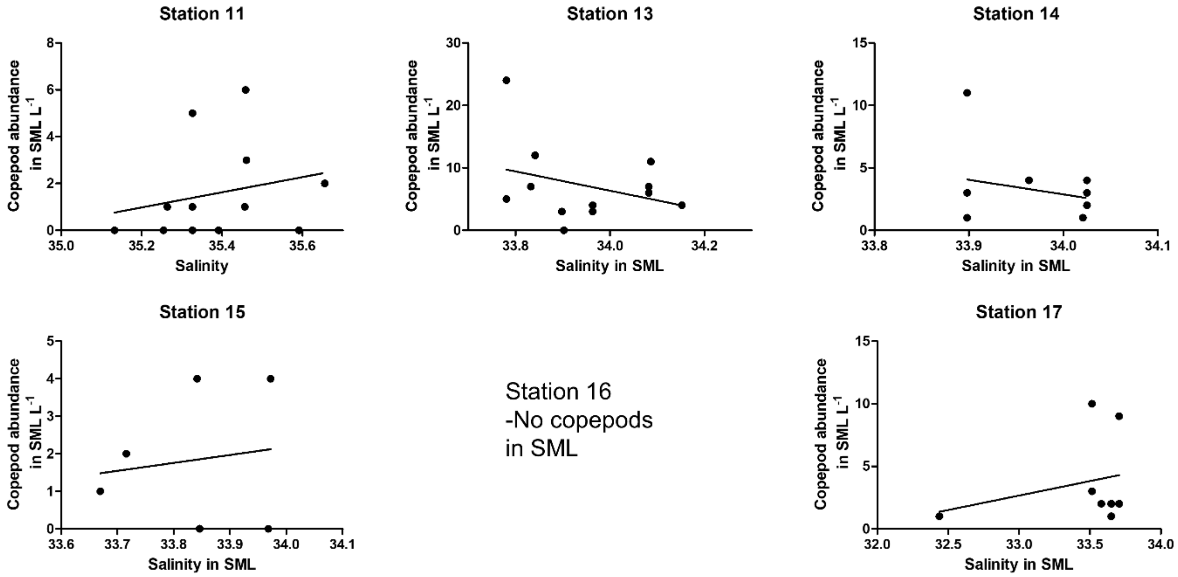

Supplementary Figure S4b: Relation of neustonic copepod abundance and salinity in SML.

Supplementary Table S3: Results from Spearman rank correlation for copepod abundance in the SML versus temperature and salinity. n.d.=not determined, n.s.=not significant

	Station number	11	13	14	15	17
**Temperature (vs copepod abundance in SML)**	Number of XY Pairs	12	12	12	7	12
	Spearman r	0	0.07195	0.1308	-0.3552	-0.2618
	p-value	1	0.8241	0.6853	0.4344	0.4111
	Significance	n.s.	n.s.	n.s.	n.s.	n.s.
**Salinity (vs copepod abundance in SML)**	Number of XY Pairs	12	12	9	6	8
	Spearman r	0.3973	-0.1996	0.1228	0.02942	0.08126
	p-value	0.2009	0.5339	0.7529	0.9959	0.8483
	Significance	n.s.	n.s.	n.s.	n.s.	n.s.

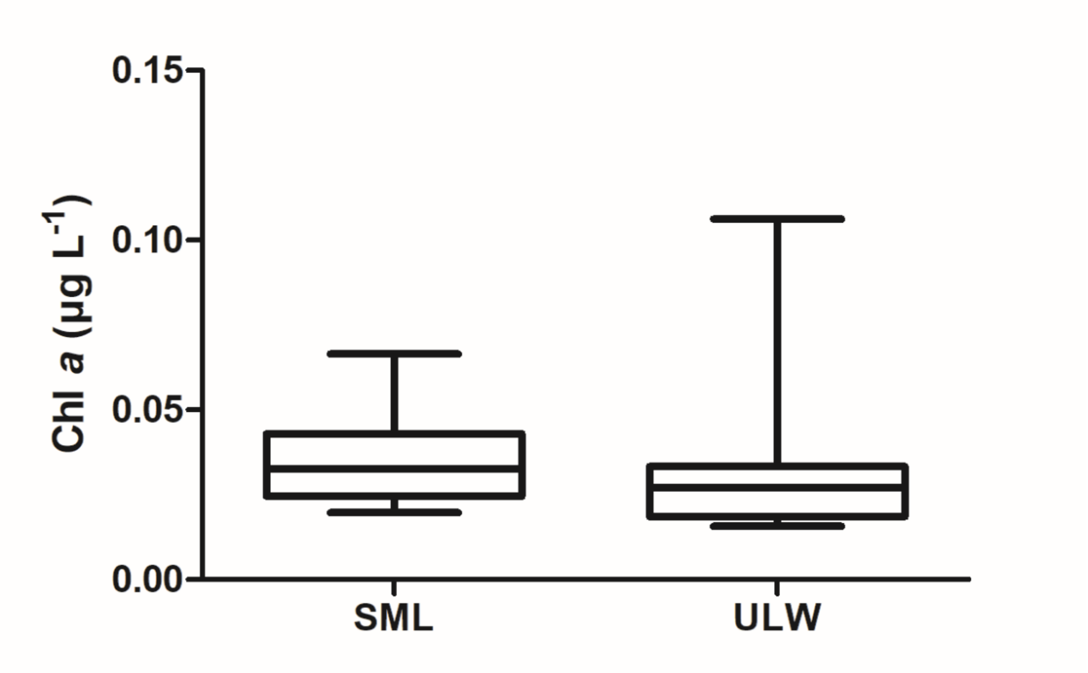

Supplementary Figure S5: Comparison of chlorophyll a concentration (µg L^{-1}) in SML and ULW

Supplementary Table S4: Statistics for chlorophyll a concentration differences in SML and ULW as determined by Mann-Whitney U-test. Whiskers represent min. and max.

**Chlorophyll a (SML vs ULW)**	Number of observations	17
	Mann-Whitney U value	103
	p-value	0.1579
	Significance	n.s.

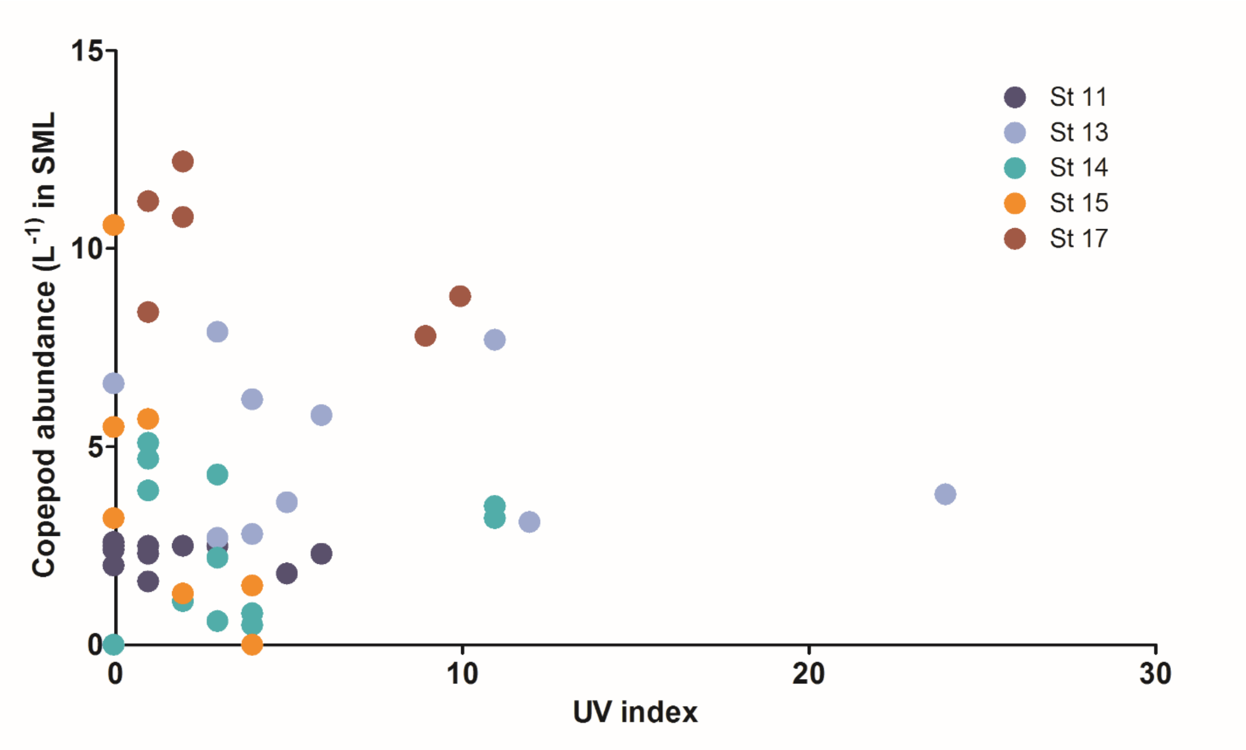

Supplementary Figure S6: Abundance of pontellid copepods (L^{-1}) in the sea-surface microlayer (SML) in relation to ultraviolet (UV) index for different stations.

Supplementary Table S5: Sample volumes of SML and ULW (containing copepods)

	S³ sampled volumes (L)	
**Station**	**SML**	**ULW**
11	12	12
13	12	10
14	12	11
15	8	7
16	12	12
17	12	12

Supplementary Table S6: Species composition of pontellid subsamples

Station	*Pontella fera* (%)	*Ivellopsis denticauda* (%)	unidentified (%)
11	0	44.4	55.6
13	33.7	0	66.3
14	29.5	0	70.5
15	n.d.	n.d.	100.0
16	n.d.	n.d.	100.0
17	38.5	0	61.5

References:

1 Schlitzer, R. (https://odv.awi.de/ (accessed 24.10.2017), 2009).
